# Evaluation of the Diagnostic Accuracy of Serum Albumin and Globulin in Pyogenic Spondylitis

**DOI:** 10.3390/jcm14176001

**Published:** 2025-08-25

**Authors:** Hideo Mitsui, Hyonmin Choe, Masashi Shimoda, Hironori Yamane, Yuta Hieda, Koki Abe, Yohei Ito, Hiroyuki Ike, Ken Kumagai, Naomi Kobayashi, Yutaka Inaba

**Affiliations:** 1 Department of Orthopedic Surgery, Yokohama City University, 3-9 Fukuura, Kanazawa-ku, Yokohama 236-0004, Japan; mt321spus@gmail.com (H.M.); shimoda.mas.wm@yokohama-cu.ac.jp (M.S.); t236074g@yokohama-cu.ac.jp (H.Y.); hieda.yut.ik@yokohama-cu.ac.jp (Y.H.); k.abe.medi@gmail.com (K.A.); youhei1021@yahoo.co.jp (Y.I.); hike@yokohama-cu.ac.jp (H.I.); kumagai@yokohama-cu.ac.jp (K.K.); yute0131@yokohama-cu.ac.jp (Y.I.); 2 Department of Orthopedic Surgery, Yokohama City University Medical Center, 4-57 Urafune-chou, Minami-ku, Yokohama 232-0024, Japan; naomik58@aol.com

**Keywords:** c-reactive protein, spondylitis, albumins, globulins, albumin–globulin ratio, biomarker

## Abstract

**Background:** Serum markers are commonly used to diagnose bone and joint infections; however, their accuracy for diagnosing pyogenic spondylitis remains unproven. This study aimed to validate the diagnostic accuracy of inflammatory, nutritional, and immunological serum markers for spinal infections and identify the most effective combinations. **Methods:** The retrospective cohort study analyzed 656 patients who visited the hospital for spinal diseases between 1 January 2004 and 31 March 2021; a total of 76 were diagnosed with pyogenic spondylitis. Blood samples were analyzed for serum albumin (Alb), total protein (TP), globulin (Glb), C-reactive protein (CRP), platelet count, white blood cell count, neutrophil count, lymphocyte count, and monocyte count. Combination markers, including albumin–globulin ratio (AGR), CRP–albumin ratio (CAR), CRP–AGR (CAGR), neutrophil–lymphocyte ratio (NLR), and platelet–lymphocyte ratio (PLR), were also evaluated. Receiver operating characteristic curves were used to determine each marker’s diagnostic performance. Furthermore, multivariate analysis was performed to examine the odds ratios. **Results:** Patients with pyogenic spondylitis showed significantly different levels in Alb (*p* < 0.0001), Glb (*p* < 0.0001), CRP (*p* < 0.0001), platelet count (*p* < 0.0001), WBC count (*p* < 0.0006), neutrophil count (*p* = 0.0019), lymphocyte count (*p* = 0.0085), AGR (*p* < 0.0001), CAR (*p* < 0.0001), CAGR (*p* < 0.0001), NLR (*p* < 0.0001), and PLR (*p* < 0.0001). CRP (AUC = 0.80) showed good diagnostic accuracy, while combination markers CAR (AUC = 0.82) and CAGR (AUC = 0.83) had the highest areas under the curve (AUC). Multivariate analysis indicated that decreased age and the presence of comorbidities (including chronic kidney disease, chronic liver disease, malignancy, or diabetes), were independent predictors of early pyogenic spondylitis (OR_age = 0.93, OR_comorbidities = 16.98, p_age = 0.0005, and p_comorbidities = 0.0001). In patients with low-inflammatory pyogenic spondylitis, significant differences were observed in TP (*p* = 0.0293), Glb (*p* = 0.0012), CRP (*p* = 0.0023), platelet count (*p* = 0.0108), AGR (*p* = 0.0044), CAR (*p* = 0.0006), CAGR (*p* = 0.0004), PLR (*p* = 0.0192), and NLR (*p* = 0.0027), with CAGR showing the highest AUC (AUC = 0.70) among them. **Conclusions:** Serum combination markers (AGR, CAGR, CAR, PLR, and NLR) showed diagnostic value for pyogenic spondylitis, with CAGR achieving the highest accuracy. In low-inflammatory pyogenic spondylitis patients (CRP ≤ 1.0 mg/dL), these markers may aid diagnosis.

## 1. Introduction

Degenerative lumbar spine diseases, characterized by lower back pain and gait disturbances, significantly impede patients’ daily activities [1]. In the differential diagnosis of low back pain and gait disturbance, neoplastic and infectious diseases are of particular importance. Particularly, infectious diseases such as pyogenic spondylitis, which are reported to be increasing in incidence because of aging populations and an increase in immunocompromised hosts, represent primary considerations when treating degenerative lumbar spine diseases [2,3,4,5]. A delayed diagnosis and misdiagnosis of pyogenic spondylitis can lead to severe spinal destruction and neurological impairment, underscoring the importance of early diagnosis [6,7]. Diagnosing pyogenic spondylitis, often accompanied by clinical symptoms, depends on imaging tests, such as magnetic resonance imaging (MRI), in addition to clinical presentation. However, diagnosis becomes challenging because of nonspecific clinical symptoms, such as isolated fever, neck pain, back pain, or lower back pain; lack of abnormalities in imaging tests; and the absence of established diagnostic flows or criteria, such as blood screening tests [8]. Serum markers are the most common and straightforward diagnostic method, and, for the examination of pyogenic spondylitis, high-sensitivity screening for infection becomes possible with elevated C-reactive protein (CRP) level and erythrocyte sedimentation rate (ESR) [9,10]. However, some pyogenic spondylitis cases are low-inflammatory, in which leukocyte count, CRP, and ESR remain unchanged even during active infection. Symptoms in patients with spinal infections are often nonspecific, leading to delayed diagnosis and prolonged time from symptom onset to diagnosis [8]. Additionally, the positive rate of blood culture has been reported as 58%, and the bacterial culture positivity rate of tissue biopsy is reported to be 77%, making the identification of the causative organism challenging [11].

In recent years, in the field of bone infections, serum markers have come to play a supportive role in the diagnosis of such challenging cases. Serum markers used in diagnosing bone and joint infections, such as nutritional markers (total protein (TP) and albumin (Alb)), blood coagulation markers (D-dimer), and immune markers (globulin (Glb), neutrophil count, lymphocyte count, monocyte count, and platelet count), have been reported to be useful [12,13]. Patients with low-inflammatory implant infections often yield false-negative results in bacterial cultures, suggesting the potential diagnostic utility of serum markers other than inflammatory markers [12]. Furthermore, combination markers, created by integrating these markers, have also attracted attention. The Alb-to-Glb ratio (AGR), which combines Alb and Glb, and the CRP-to-Alb ratio (CAR), which combines CRP and Alb, have been reported to be useful for diagnosing infections after total hip arthroplasty [12]. The combination marker CRP–AGR (CAGR), which integrates Alb, Glb, and CRP, has been reported to be useful for diagnosing infections after joint arthroplasty, as it reflects systemic inflammatory responses well and functions as a valuable diagnostic indicator of inflammation [14,15]. In addition, the neutrophil-to-lymphocyte ratio (NLR), which combines neutrophils and lymphocytes, and platelet-to-lymphocyte ratio (PLR), which combines platelets and lymphocytes, have been reported to be useful for the early detection of postoperative infections after joint arthroplasty [16,17,18]. NLR has been reported to be useful for the early diagnosis of postoperative infections after spinal fusion surgery, thereby enhancing diagnostic capability [19]. However, no evidence has been reported regarding the usefulness or accuracy of these serum markers in diagnosing pyogenic spondylitis.

This study aimed to investigate the utility of nutritional and immune markers, as well as combination markers derived from them, in diagnosing pyogenic spondylitis and to examine whether their combined use with conventional serum markers contributes to improving diagnostic accuracy in patients with pyogenic spondylitis.

## 2. Materials and Methods

### 2.1. Patient Enrollment

**Figure 1:**
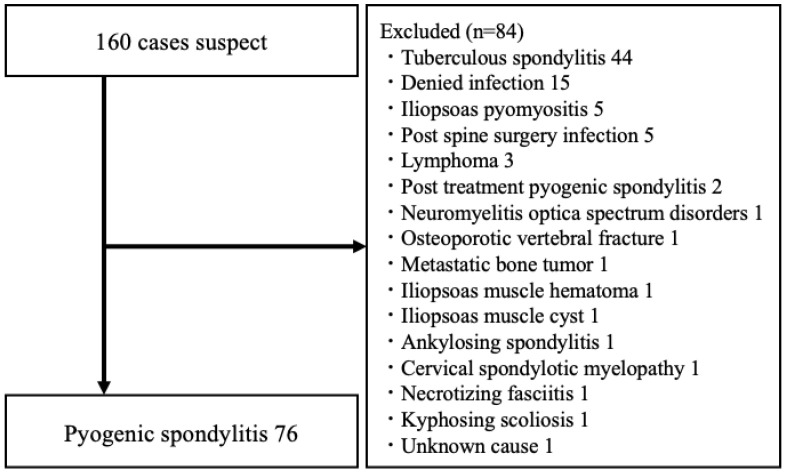
Patient enrollment flow diagram.

This retrospective cohort study included 656 patients who visited our hospital, between 1 January 2004 and 31 March 2021, for the treatment of spinal diseases. This research was approved by affiliated institutions and the institutional review board (approval number: B210500004). Consent to participate, and for publication, was obtained by providing the opt-out information on the homepage of our hospital. Infection was suspected in 160 patients who presented with clinical symptoms such as fever, neck pain, and lumbar back pain. Imaging tests, including radiography and computed tomography (CT), revealed vertebral endplate irregularities, absorption images, areas of bone destruction, reduced intervertebral disk height, and elevation of serum inflammatory markers (white blood cell [WBC] count >8600/μL, CRP level >0.15 mg/dL). Bacterial culture pathology tests were performed using blood cultures obtained from patients or tissues acquired by intervertebral disk puncture. Cases with positive results were diagnosed as infectious. Even if tissue examinations were negative, cases were diagnosed as infectious if T1-weighted image low-signal areas, T2-weighted image high-signal areas, and short tau inversion recovery (STIR) method high-signal areas were observed in the intervertebral disks and vertebral endplates on MRI without clear evidence of infection elsewhere. In this retrospective investigation, 76 patients were diagnosed with pyogenic spondylitis and 84 patients were diagnosed with non-pyogenic spondylitis, respectively (Figure 1). Among the 84 patients diagnosed with non-pyogenic spondylitis, 44 had tuberculous spondylitis, 15 showed nonspecific inflammation, 5 had iliopsoas abscesses, 5 had a history of post-spinal fusion surgery infections, 2 patients each had malignant lymphoma and a history of pyogenic spondylitis, and 1 patient each had optic neuritis, osteoporotic vertebral fractures, metastatic bone tumors, iliopsoas muscle hemorrhage after surgery, diffuse large B-cell lymphoma, iliopsoas muscle cysts, post-decompression surgery infection, ankylosing spondylitis, cervical myelopathy, necrotizing fasciitis, degenerative scoliosis, and an unclear disease. Serum markers were investigated in the 76 patients with pyogenic spondylitis. Patients had not been administered antibiotics before undergoing blood tests and treatment. Serum markers were also examined in a control group of 531 patients diagnosed with lumbar spinal stenosis who underwent surgical treatment during the same period without any infectious signs.

### 2.2. Acquisition of Data

Patient data collected in this study included age, sex, and the presence of comorbidities. Comorbidities were defined as present if the patient had any of the following conditions: chronic kidney disease (CKD), chronic liver disease, malignancy, or diabetes mellitus.

### 2.3. Serum Biomarkers

Serum markers investigated in this study included CRP as an inflammatory marker; TP, Alb, and Glb as nutritional markers; and platelet, WBC, neutrophil, lymphocyte, and monocyte counts as immune markers. Additionally, combination markers, such as Alb–Glb ratio (AGR), CRP–Alb ratio (CAR), CRP–AGR (CAGR), neutrophil-to-lymphocyte ratio (NLR), and platelet-to-lymphocyte ratio (PLR) were examined. Among the 76 patients with pyogenic spondylitis, patients with a CRP level ≤1.0 mg/dL were defined as having low-inflammation spinal infections, and the diagnostic accuracy of serum markers in these patients was investigated. Overall, 35 patients with pyogenic spondylitis had low inflammation. Investigation parameters included WBC count, neutrophil count, and CRP as inflammatory markers, along with TP, Alb, Glb, platelet count, lymphocyte count, and monocyte count as immune markers, consistent with those examined in the group of 76 patients with pyogenic spondylitis. Additionally, the AGR, CAR, CAGR, NLR, and PLR were investigated. Among the patients with pyogenic spondylitis, 43 patients were culture positive. The diagnostic accuracy of serum markers was also investigated specifically in these culture-positive patients.

### 2.4. Statistical Analysis

The normality of the data was assessed using the Shapiro–Wilk test. Background characteristics of patients in the pyogenic spondylitis and lumbar spinal stenosis groups, as well as the results of blood tests, were compared using the Mann–Whitney U test. Receiver operating characteristic (ROC) curves were constructed for blood test results, and the area under the curve (AUC) was calculated to verify diagnostic accuracy for pyogenic spondylitis. Cut-off values were identified using the Youden index, and calculations were performed to measure sensitivity, specificity, positive predictive value, negative predictive value, accuracy, positive likelihood ratio, and negative likelihood ratio for each serum marker. A three-groups comparison was performed using ANOVA followed by Tukey’s post hoc test. All statistical analyses were performed using JMP Pro 18 (SAS Institute, Tokyo, Japan). Statistical significance was set at *p* < 0.05.

## 3. Results

The analysis in this study included 607 patients. Among them, 76 had pyogenic spondylitis, of which 35 (46.1%) were classified as low-inflammatory pyogenic spondylitis. There were 43 culture-positive cases (56.6%) among the patients with pyogenic spondylitis. The control group consisted of 531 patients. None of the serum laboratory data followed a normal distribution.

### 3.1. Comparison Between the Pyogenic Spondylitis and Control Groups

Patients in the pyogenic spondylitis group were significantly younger than those in the control group (65.9 years vs. 71.2 years, *p* < 0.0001), and a significantly higher proportion of male patients was observed in the pyogenic spondylitis group compared to that in the lumbar spinal stenosis group (68% vs. 54%, *p* = 0.0191). Furthermore, comorbidities were significantly more common in the pyogenic spondylitis group than in the control group (48.7% vs. 3.8%, *p* < 0.0001) (Table 1). Significant differences were observed between the pyogenic spondylitis and control groups in Alb (*p* < 0.0001), Glb (*p* < 0.0001), CRP (*p* < 0.0001), platelet count (*p* < 0.0001), WBC count (*p* = 0.0006), neutrophil count (*p* = 0.0019), lymphocyte count (*p* = 0.0085), AGR (*p* < 0.0001), CAR (*p* < 0.0001), CAGR (*p* < 0.0001), NLR (*p* < 0.0001), and PLR (*p* < 0.0001), whereas no significant differences were observed in TP (*p* = 0.3003) and monocyte counts (*p* = 0.5268) (Table 2).

### 3.2. Diagnostic Accuracy of Serum Biomarkers for Pyogenic Spondylitis

**Figure 2:**
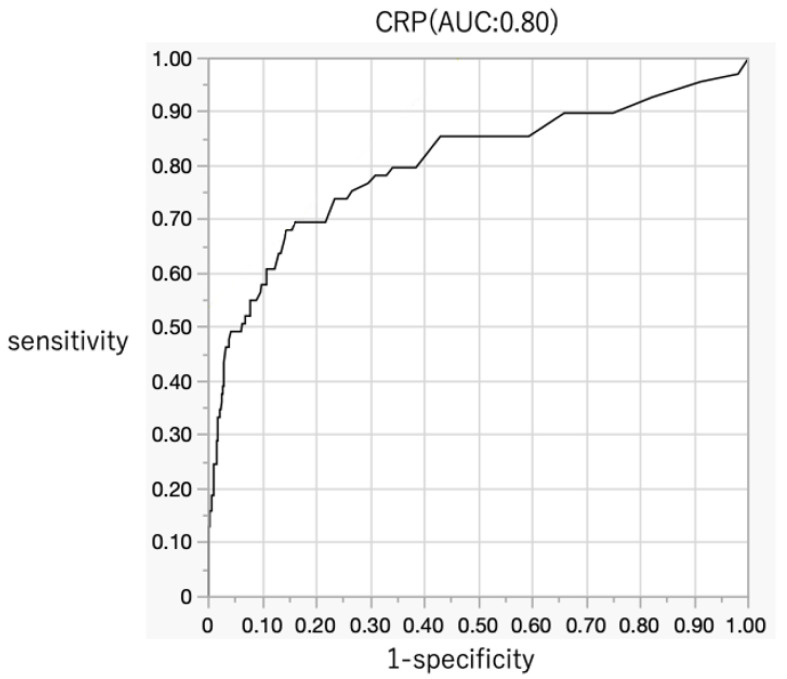
Diagnostic accuracy of CRP for pyogenic spondylitis (AUC: 0.80). AUC, area under the curve; CRP, C-reactive protein.

The AUC for CRP in the ROC curve was 0.80 (95% CI = 0.73–0.86), indicating high accuracy as a blood diagnostic marker for pyogenic spondylitis (Figure 2). However, the AUCs for Alb, Glb, platelet count, WBC count, neutrophil count, and lymphocyte count were 0.70 (0.61–0.78), 0.77 (0.69–0.84), 0.68 (0.61–0.75), 0.62 (0.55–0.69), 0.63 (0.54–0.71), and 0.60 (0.53–0.67), respectively, suggesting lower diagnostic accuracy compared to that for CRP (Table 3).

### 3.3. Diagnostic Accuracy of Combination Biomarkers for Pyogenic Spondylitis

**Figure 3:**
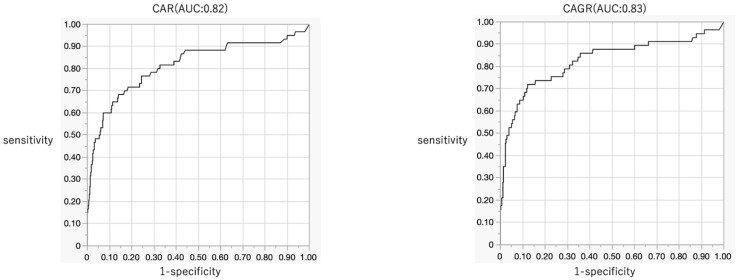
Diagnostic accuracy of CAR and CAGR for pyogenic spondylitis (AUC: 0.82 and 0.83, respectively). AUC, area under the curve; CAR, CRP-albumin ratio; AGR, albumin–globulin ratio; CAGR, CRP–AGR.

Among the combination biomarkers, the AUCs for CAR and CAGR were 0.82 (0.73–0.88) and 0.83 (0.74–0.89), respectively (CAR: sensitivity 0.6833, specificity 0.8587 at a cut-off value of 0.09; CAGR: sensitivity 0.7193, specificity 0.877 at a cut-off value of 0.28), indicating high diagnostic accuracy. Conversely, the AUCs for AGR, NLR, and PLR were 0.77 (0.68–0.84), 0.69 (0.61–0.75), and 0.67 (0.59–0.75), respectively, indicating lower diagnostic accuracy compared to that for CAR and CAGR (Figure 3, Table 3).

### 3.4. Multivariate Analysis for Pyogenic Spondylitis

Multivariate analysis indicated that decreased age and the presence of comorbidities (including chronic kidney disease, chronic liver disease, malignancy, or diabetes mellitus), were independent predictors of early pyogenic spondylitis (Table 4, OR_age = 0.93, OR_comorbidities = 16.98, p_age = 0.0005, and p_comorbidities < 0.0001).

### 3.5. Comparison Between the Low-Inflammatory Pyogenic Spondylitis and Control Groups

**Figure 4:**
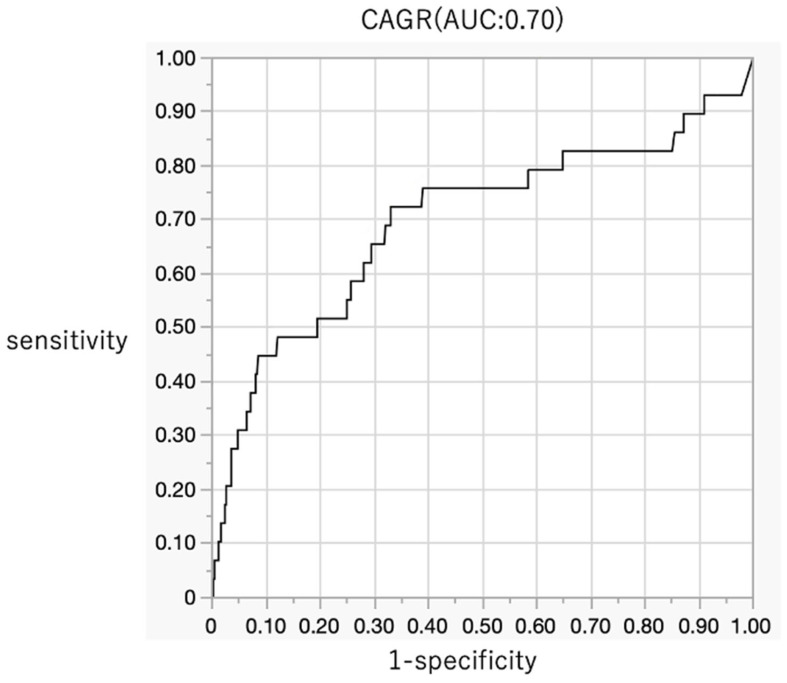
Diagnostic accuracy of CAGR for the low-inflammatory pyogenic spondylitis (AUC: 0.70). AUC, area under the curve; CRP-albumin ratio; AGR, albumin–globulin ratio; CAGR, CRP–AGR.

In comparisons involving the low-inflammatory pyogenic spondylitis group, significant differences were observed in TP (*p* = 0.0293), Glb (*p* = 0.0012), CRP (*p* = 0.0023), platelet count (*p* = 0.0108), AGR (*p* = 0.0044), CAR (*p* = 0.0006), CAGR (*p* = 0.0004), PLR (*p* = 0.0192), and NLR (*p* = 0.0027). No significant differences were observed in Alb (*p* = 0.1193), WBC count (*p* = 0.6551), neutrophil count (*p* = 0.1254), lymphocyte count (*p* = 0.0541), and monocyte count (*p* = 0.2099) (Table 5). Among the serum markers evaluated in the low-inflammatory pyogenic spondylitis group, CAGR demonstrated the highest AUC (0.69711 (95% CI = 0.56–0.80)) with a cut-off value of 0.08, sensitivity of 0.7241, and specificity of 0.6698 (Figure 4, Table 6).

### 3.6. Comparison Between the Culture-Positive Pyogenic Spondylitis and Control Groups

In comparisons involving the culture-positive pyogenic spondylitis group, significant differences were observed in Alb (*p* < 0.0001), Glb (*p* < 0.0001), CRP (*p* < 0.0001), platelet count (*p* < 0.0001), WBC count (*p* = 0.0004), neutrophil count (*p* = 0.0018), lymphocyte count (*p* = 0.0297), AGR (*p* < 0.0001), CAR (*p* < 0.0001), CAGR (*p* < 0.0001), NLR (*p* < 0.0001), and PLR (*p* < 0.0001), whereas no significant differences were observed in TP (*p* = 0.3285) and monocyte counts (*p* = 0.4961) (Table 7). Among the serum markers evaluated in the culture-positive pyogenic spondylitis group, CAGR demonstrated the highest AUC (0.8422 (95% CI = 0.73–0.91)) with a cut-off value of 0.3, sensitivity of 0.7576, and specificity of 0.877 (Table 8).

A three-groups comparison was performed among the culture-positive group, culture-negative group, and control group. Significant differences in Alb, Glb, CRP, AGR, CAR, and CAGR were observed between the culture-positive group and the control group (*p* < 0.0001, respectively), as well as between the culture-negative group and the control group (*p* < 0.0001, respectively).

## 4. Discussion

This study aimed to determine whether combining conventional serum markers with nutritional and immunological markers can improve the screening accuracy for pyogenic spondylitis, and to evaluate whether albumin (Alb) as a nutritional marker, globulin (Glb) as an immunological marker, and their combination markers function as effective diagnostic indicators. Alb and Glb are commonly used to assess nutritional and immune status and as markers of infection and inflammation. Alb levels decrease owing to consumption during tissue repair caused by inflammation, tumors, trauma, or surgery [20,21]. In contrast, Glb, calculated by subtracting Alb from TP, has been used to evaluate conditions such as infections, collagen diseases, and cirrhosis. Produced by plasma cells in lymphoid tissues, Glb plays a crucial role in immune responses and increases during inflammation [22]. This immune response involves interleukin-6 secretion by activated macrophages following infection, which inhibits Alb production in the liver while stimulating immunoglobulin production, resulting in reduced Alb and elevated Glb levels [23,24,25,26]. Therefore, elevated Glb reflects increased antibody production during infection, allowing Glb measurement to assess immune and inflammatory status.

The infection-induced decrease in Alb and increase in Glb leads to a lowered AGR. AGR, representing the Alb–Glb ratio, has been reported to decline during infections and has been proposed as a prognostic marker in various diseases [25,26,27,28]. Although TP reflects the sum of Alb and Glb, no significant difference in TP was observed between the pyogenic spondylitis and control groups in this study. However, decreased Alb and increased Glb levels associated with pyogenic spondylitis were clearly reflected by a reduced AGR. Additionally, combination markers such as CAR and CAGR, which include CRP, Alb, and Glb, demonstrated high diagnostic accuracy. Among them, CAGR showed the highest accuracy, suggesting that evaluating Alb and Glb levels contributes meaningfully to diagnosing pyogenic spondylitis.

Previous studies have identified high sensitivity for WBC, CRP, and ESR in detecting pyogenic spondylitis and other infections [9,10]. Most recent research has highlighted the diagnostic value of platelet, neutrophil, lymphocyte, and monocyte counts in prosthetic joint infections [12,13]. Although WBC elevations represent an immune response to bacterial invasion, increased platelet counts result from thrombopoietin production—a liver-mediated acute-phase reaction to inflammation [29]. This study demonstrated that AGR, in addition to CRP, served as a valuable diagnostic marker for spinal infections, including pyogenic spondylitis.

In addition to CRP and WBC, this study demonstrated that Alb, Glb, and combination markers including AGR, CAGR, CAR, PLR, and NLR serve as valuable diagnostic markers for pyogenic spondylitis. Furthermore, it was revealed that younger patients with comorbidities exhibit an increased risk of developing pyogenic spondylitis. In patients presenting symptoms suggestive of pyogenic spondylitis, focusing on Alb, Glb, and these combination markers may enhance the potential for early diagnosis. Pyogenic spondylitis does not always present with elevated CRP levels; in this study, 35 patients had CRP levels below 1.0 mg/dL, suggesting the presence of a subset with low-inflammatory pyogenic spondylitis. In these cases, the combination marker CAGR, which integrates CRP and AGR, demonstrated superior diagnostic accuracy and complemented the limitations of CRP alone in avoiding missed diagnoses. When CRP values are low or within the normal range, early infection should be considered, prompting timely imaging with high diagnostic capability, such as MRI, and early biopsy planning for definitive diagnosis. In settings where access to CT, MRI, or specialist-performed biopsy is limited, reliance on these serum markers can facilitate earlier intervention. Regarding the variables Alb, Glb, CRP, AGR, CAR, and CAGR, a three-groups comparison among the culture-positive, culture-negative, and control group revealed that both the culture-positive and culture-negative groups showed significant differences compared to the control group. These results suggest that even in culture-negative cases, these markers may be useful as adjuncts in the diagnosis of pyogenic spondylitis. However, the AUC of CAGR in patients with CRP ≤1.0 mg/dL was approximately 0.7, indicating that these markers should be regarded as supplementary rather than standalone diagnostic tools. Importantly, these combination markers are automatically calculated from routinely measured Alb, Glb, and complete blood count results, incurring no additional cost or time, which enhances their clinical applicability.

This study had several limitations. First, the investigation was conducted at a single center, which may have limited the generalizability of the findings. Validation through multicenter studies would be necessary to confirm the usefulness of the identified markers. Second, the background characteristics of patients with spinal infections varied widely, including individuals with diabetes, immunocompromised conditions, cancer, and those without underlying health issues. Future studies should consider analyzing populations with more consistent background factors. Third, although the analysis grouped all patients under the broad category of spinal infections, including pyogenic spondylitis, tuberculous spondylitis, and psoas abscess, the diagnostic performance of markers may differ by disease type. Therefore, separate analyses by infection category would be desirable in future research. Fourth, the diagnosis of culture-negative patients is based on MRI findings, which may lead to overdiagnosis or include non-infectious conditions that mimic purulent spondylitis. This diagnostic uncertainty can affect the specificity of serum markers and should, therefore, be considered when interpreting the results. Finally, the control group consisted solely of elderly patients who had undergone surgery for lumbar spinal canal stenosis. Elderly patients often have comorbidities, which may be accompanied by inflammatory conditions that could affect serum markers. In this study, all surgical patients were confirmed to be free of infection preoperatively based on clinical findings, laboratory results, and imaging studies. However, we believe that including healthy individuals in future studies would allow for more refined comparative research.

## 5. Conclusions

Alb, Glb, and their combination markers (AGR, CAGR, CAR, PLR, and NLR) demonstrated diagnostic utility for pyogenic spondylitis alongside CRP and WBC, with CAGR showing the highest accuracy. Particularly in low-inflammatory patients (CRP ≤ 1.0 mg/dL), combination markers such as CAGR can be useful in diagnosing pyogenic spondylitis. While these markers are useful as adjunctive tools, further multicenter studies are needed to validate these findings and assess disease-specific performance.

## Figures and Tables

**Table 1:** Group differences in demographic characteristics.

Group	Pyogenic Spondylitis	Control	*p* Value
Number of patients	76	531	
Age; mean (range)	65 (38–86)	71 (29–96)	<0.0001
Proportion of male patients	68.4%	54.1%	0.0191
Comorbidities	37 (48.7%)	20 (3.8%)	<0.0001

**Table 2:** Mean (standard deviation) values of serum markers for pyogenic spondylitis.

Category	Markers	Pyogenic Spondylitis	Control	*p* Value
Nutritional markers	Albumin, g/dL	3.6 (0.9)	4.2 (0.4)	<0.0001
	Total protein, g/dL	7.1 (0.8)	7.1 (0.5)	0.3003
	Globulin, g/dL	3.5 (0.8)	2.9 (0.4)	<0.0001
	AGR	1.1 (0.5)	1.5 (0.3)	<0.0001
Inflammatory markers	C-reactive protein, mg/dL	4.7 (8.8)	0.3 (0.8)	<0.0001
	CAR	1.8 (4)	0.1 (0.2)	<0.0001
	CAGR	7 (15)	0.2 (0.8)	<0.0001
Hematologic markers	White blood cell count, /μL	7924 (3606)	6469 (1942)	0.0006
	N count, /μL	5182 (3242)	3985 (1576)	0.0019
	L count, /μL	1463 (568)	1683 (638)	0.0085
	M count, /μL	529 (258)	480 (176)	0.5268
	Platelet count, 104/μL	29.4 (12.9)	22.6 (6.1)	<0.0001
	NLR	4.1 (3.8)	2.6 (2.3)	<0.0001
	PLR	250.4 (190.9)	158.6 (90.1)	<0.0001

AGR, albumin/globulin ratio; CAR, CRP-albumin ratio; CAGR, CRP–AGR; N, neutrophil; L, lymphocyte; M, monocyte; NLR, neutrophil–lymphocyte ratio; PLR, platelet–lymphocyte ratio.

**Table 3:** Diagnostic accuracy of serum markers for pyogenic spondylitis.

Category	Markers	AUC (95% CI)	Cut-Off	Sensitivity	Specificity	PPV	NPV
Nutritional	Albumin, g/dL	0.70 (0.61–0.78)	3.7 ^1^	0.52	0.88	0.39	0.93
	Globulin, g/dL	0.77 (0.69–0.84)	3.2 ^1^	0.66	0.81	0.31	0.95
	AGR	0.77 (0.68–0.84)	1.2 ^1^	0.61	0.87	0.38	0.94
Inflammatory	C-reactive protein, mg/dL	0.80 (0.73–0.86)	0.3 ^1^	0.68	0.86	0.38	0.95
	CAR	0.82 (0.73–0.88)	0.1 ^1^	0.68	0.86	0.39	0.95
	CAGR	0.83 (0.74–0.89)	0.3 ^1^	0.72	0.88	0.43	0.96
Hematologic	White blood cell count, /μL	0.62 (0.55–0.69)	7800 ^1^	0.42	0.80	0.23	0.91
	N count, /μL	0.63 (0.54–0.71)	3680 ^1^	0.73	0.51	0.17	0.93
	L count, /μL	0.60 (0.53–0.67)	1700 ^1^	0.72	0.47	0.18	0.91
	Platelet count, 10^4^/μL	0.68 (0.61–0.75)	27.0 ^1^	0.53	0.78	0.25	0.92
	NLR	0.69 (0.61–0.75)	2.0 ^1^	0.84	0.48	0.18	0.96
	PLR	0.67 (0.59–0.75)	208 ^1^	0.51	0.83	0.33	0.91

AUC, area under curve; CI, confidence interval; PPV, positive predictive value; NPV, negative predictive value; AGR, albumin/globulin ratio; CAR, CRP-albumin ratio; CAGR, CRP–AGR; N, neutrophil; L, lymphocyte; NLR, neutrophil–lymphocyte ratio; PLR, platelet–lymphocyte ratio. ^1^ Youden’s index.

**Table 4:** Multivariate analysis for pyogenic spondylitis.

Category	Variable	OR	95% CI	*p* Value
	Age	0.93	0.90–0.97	0.0005
	Comorbidities	16.98	6.31–45.73	<0.0001
Nutritional markers	Albumin, g/dL	0.24	0.03–2.12	0.1974
	Globulin, g/dL	6.1	0.43–97.02	0.1905
	AGR	5.99	0.01–2395.08	0.5796
Inflammatory markers	C-reactive protein, mg/dL	0.63	0.13–1.32	0.4404
	CAGR	1.53	0.91–5.73	0.3283
Hematologic markers	NLR	1.06	0.85–1.26	0.5843
	PLR	1.00	0.995–1.003	0.8819

OR, odds ratio; CI, confidence interval; AGR, albumin/globulin ratio; CAGR, CRP–AGR; NLR, neutrophil–lymphocyte ratio; PLR, platelet–lymphocyte ratio.

**Table 5:** Mean (standard deviation) values of serum markers for low-inflammatory pyogenic spondylitis.

Category	Markers	Low-Inflammatory Pyogenic Spondylitis	Control	*p* Value
Nutritional markers	Albumin, g/dL	4.0 (0.8)	4.2 (0.4)	0.1193
	Total protein, g/dL	7.3 (0.6)	7.1 (0.5)	0.0293
	Globulin, g/dL	3.3 (1.0)	2.8 (0.4)	0.0012
	AGR	1.3 (0.4)	1.5 (0.3)	0.0044
Inflammatory markers	C-reactive protein, mg/dL	0.24 (0.22)	0.13 (0.18)	0.0023
	CAR	0.073 (0.069)	0.03 (0.05)	0.0006
	CAGR	0.29 (0.33)	0.1 (0.2)	0.0004
Hematologic markers	White blood cell count, /μL	6303 (1622)	6380 (1890)	0.6551
	N count, /μL	4149 (1275)	3907 (1514)	0.1254
	L count, /μL	1482 (527)	1698 (633)	0.0541
	M count, /μL	437 (152)	473 (172)	0.2099
	Platelet count, 10^4^/μL	25.5 (8.6)	22.5 (6)	0.0108
	NLR	8759 (6743)	85,636 (87,653)	0.0027
	PLR	193.5 (99.7)	154.4 (79.7)	0.0192

AGR, albumin/globulin ratio; CAR, CRP-albumin ratio; CAGR, CRP–AGR; N, neutrophil; L, lymphocyte; M, monocyte; NLR, neutrophil–lymphocyte ratio; PLR, platelet–lymphocyte ratio.

**Table 6:** Diagnostic accuracy of serum markers for low-inflammatory pyogenic spondylitis.

Category	Markers	AUC (95% CI)	Cut-Off	Sensitivity	Specificity	PPV	NPV
Nutritional	Total protein, g/dL	0.61 (0.50–0.72)	7.3 ^1^	0.63	0.65	0.10	0.96
	Globulin, g/dL	0.68 (0.56–0.78)	3.0 ^1^	0.66	0.63	0.11	0.96
	AGR	0.66 (0.53–0.77)	1.2 ^1^	0.41	0.89	0.20	0.96
Inflammatory	C-reactive protein, mg/dL	0.65 (0.54–0.75)	0.1 ^1^	0.71	0.60	0.11	0.97
	CAR	0.68 (0.56–0.79)	0.02 ^1^	0.77	0.58	0.12	0.97
	CAGR	0.70 (0.56–0.80)	0.1 ^1^	0.72	0.67	0.13	0.97
Hematologic	Platelet count, 10^4^/μL	0.63 (0.53–0.72)	23.3 ^1^	0.66	0.57	0.10	0.96
	NLR	0.66 (0.58–0.74)	2.0 ^1^	0.81	0.49	0.11	0.97
	PLR	0.62 (0.51–0.72)	209 ^1^	0.42	0.85	0.19	0.99

AUC, area under curve; CI, confidence interval; PPV, positive predictive value; NPV, negative predictive value; AGR, albumin/globulin ratio; CAR, CRP-albumin ratio; CAGR, CRP–AGR; NLR, neutrophil–lymphocyte ratio; PLR, platelet–lymphocyte ratio. ^1^ Youden’s index.

**Table 7:** Mean (standard deviation) values of serum markers for culture-positive pyogenic spondylitis.

Category	Markers	Culture-Positive Pyogenic Spondylitis	Control	*p* Value
Nutritional markers	Albumin, g/dL	3.5 (0.9)	4.2 (0.4)	<0.0001
	Total protein, g/dL	7.2 (0.7)	7.1 (0.5)	0.3285
	Globulin, g/dL	3.7 (0.9)	2.9 (0.4)	<0.0001
	AGR	1.0 (0.4)	1.5 (0.3)	<0.0001
Inflammatory markers	C-reactive protein, mg/dL	5.3 (9.9)	0.3 (0.8)	<0.0001
	CAR	1.8 (3.5)	0.1 (0.2)	<0.0001
	CAGR	7.7 (15)	0.2 (0.8)	<0.0001
Hematologic markers	White blood cell count, /μL	8272 (3774)	6469 (1942)	0.0004
	N count, /μL	5633 (3772)	3985 (1576)	0.0018
	L count, /μL	1450 (549)	1683 (638)	0.0297
	M count, /μL	547 (280)	480 (176)	0.4961
	Platelet count, 10^4^/μL	31.5 (15.1)	22.6 (6.1)	<0.0001
	NLR	4.7 (4.6)	2.6 (2.3)	<0.0001
	PLR	276.2 (223.8)	158.6 (90.1)	<0.0001

AGR, albumin/globulin ratio; CAR, CRP-albumin ratio; CAGR, CRP–AGR; N, neutrophil; L, lymphocyte; M, monocyte; NLR, neutrophil–lymphocyte ratio; PLR, platelet–lymphocyte ratio.

**Table 8:** Diagnostic accuracy of serum markers for culture-positive pyogenic spondylitis.

Category	Markers	AUC (95% CI)	Cut-Off	Sensitivity	Specificity	PPV	NPV
Nutritional	Albumin, g/dL	0.75 (0.63–0.84)	3.7 ^1^	0.62	0.88	0.31	0.97
	Globulin, g/dL	0.81 (0.71–0.89)	3.5 ^1^	0.59	0.91	0.34	0.97
	AGR	0.81 (0.70–0.89)	0.1 ^1^	0.71	0.88	0.32	0.98
Inflammatory	C-reactive protein, mg/dL	0.84(0.74–0.90)	0.4 ^1^	0.76	0.86	0.29	0.98
	CAR	0.84 (0.73–0.91)	0.1 ^1^	0.74	0.86	0.30	0.97
	CAGR	0.84 (0.73–0.91)	0.3 ^1^	0.76	0.88	0.32	0.98
Hematologic	White blood cell count, /μL	0.66 (0.56–0.75)	7800 ^1^	0.49	0.80	0.16	0.95
	N count, /μL	0.66 (0.55–0.76)	5421 ^1^	0.42	0.86	0.20	0.95
	L count, /μL	0.60 (0.51–0.69)	1588 ^1^	0.66	0.53	0.12	0.94
	Platelet count, 10^4^/μL	0.71 (0.61–0.79)	30.5 ^1^	0.40	0.91	0.26	0.95
	NLR	0.70 (0.61–0.78)	2.2 ^1^	0.82	0.52	0.12	0.97
	PLR	0.69 (0.58–0.78)	195 ^1^	0.59	0.77	0.20	0.95

AUC, area under curve; CI, confidence interval; PPV, positive predictive value; NPV, negative predictive value; AGR, albumin/globulin ratio; CAR, CRP-albumin ratio; CAGR, CRP–AGR; N, neutrophil; L, lymphocyte; NLR, neutrophil–lymphocyte ratio; PLR, platelet–lymphocyte ratio. ^1^ Youden’s index.

## Data Availability

Requests for access should be directed to the corresponding author.
